# Increased signaling by the autism-related Engrailed-2 protein enhances dendritic branching and spine density, alters synaptic structural matching, and exaggerates protein synthesis

**DOI:** 10.1371/journal.pone.0181350

**Published:** 2017-08-15

**Authors:** Asma Soltani, Solène Lebrun, Gilles Carpentier, Giulia Zunino, Sandrine Chantepie, Auriane Maïza, Yuri Bozzi, Claire Desnos, François Darchen, Olivier Stettler

**Affiliations:** 1 UMR 8250, Centre National de la Recherche Scientifique, Université Paris Descartes, Sorbonne Paris Cité, Paris, France; 2 Laboratoire Croissance, Réparation et Régénération Tissulaires (CRRET), EA 4397 / ERL 9215, Centre National de la Recherche Scientifique, Université Paris Est Créteil, Créteil, France; 3 Centre for Integrative Biology, University of Trento, Trento, Italy; Bilkent University, TURKEY

## Abstract

*Engrailed 1* (*En1*) and 2 (*En2*) code for closely related homeoproteins acting as transcription factors and as signaling molecules that contribute to midbrain and hindbrain patterning, to development and maintenance of monoaminergic pathways, and to retinotectal wiring. *En2* has been suggested to be an autism susceptibility gene and individuals with autism display an overexpression of this homeogene but the mechanisms remain unclear. We addressed in the present study the effect of exogenously added En2 on the morphology of hippocampal cells that normally express only low levels of Engrailed proteins. By means of RT-qPCR, we confirmed that En1 and En2 were expressed at low levels in hippocampus and hippocampal neurons, and observed a pronounced decrease in En2 expression at birth and during the first postnatal week, a period characterized by intense synaptogenesis. To address a putative effect of Engrailed in dendritogenesis or synaptogenesis, we added recombinant En1 or En2 proteins to hippocampal cell cultures. Both En1 and En2 treatment increased the complexity of the dendritic tree of glutamatergic neurons, but only En2 increased that of GABAergic cells. En1 increased the density of dendritic spines both *in vitro* and *in vivo*. En2 had similar but less pronounced effect on spine density. The number of mature synapses remained unchanged upon En1 treatment but was reduced by En2 treatment, as well as the area of post-synaptic densities. Finally, both En1 and En2 elevated mTORC1 activity and protein synthesis in hippocampal cells, suggesting that some effects of Engrailed proteins may require mRNA translation. Our results indicate that Engrailed proteins can play, even at low concentrations, an active role in the morphogenesis of hippocampal cells. Further, they emphasize the over-regulation of GABA cell morphology and the vulnerability of excitatory synapses in a pathological context of En2 overexpression.

## Introduction

Autism spectrum disorders (ASDs) comprise heterogeneous neurodevelopmental disorders characterized by significant social and communication deficits and by repetitive and stereotyped behaviors. In syndromic ASDs (e.g., fragile X syndrome, Angelman syndrome, tuberous sclerosis), autism is one component of a broader phenotype [1]. ASDs manifest at key steps of brain development when sensory experience is modifying synapse maturation and when inhibitory synapses become functional [2]. Targeted mutations of several susceptibility genes for ASDs with seemingly unrelated functions often lead to similar abnormalities of the development of dendrites and synapses of excitatory glutamatergic neurons in mice [3]. This phenotypical convergence suggests that ASD may originate in altered synaptic development leading to imbalance between excitation and inhibition (E/I), and defects in brain connectivity [2–4]. In this respect, many genes linked to ASDs have been shown to control the morphology and signaling of dendrites and dendritic spines of excitatory neurons [3], by encoding synaptic adhesion or scaffolding proteins (e.g., Shank3, Neuregulin, Mecp2, Neuroligin) [3], or molecules regulating protein synthesis (e.g., PTEN, TSC1/2, NF1, and FMRP) [3,5].

Several human studies have raised the hypothesis of an association of *Engrailed 2* (En2) with ASD [6–9]. Significantly in this respect were the observations that levels of En2 protein were elevated in the brain of individuals with ASD compared to unaffected controls [8, 9]. However, the functional link between excessive levels of En2 and ASDs is still poorly understood [9]. For instance, it is not known if En2 or its homolog En1 can affect dendritic spines and synapses. Engrailed (En, which collectively refers to En1 and En2) belongs to a large family of homeodomain-containing transcription factors and has various regulatory roles at different stages of development involving transcriptional and translational activities [10,11]. Developmental studies have shown that En transcriptional activity is required for segmentation and regionalization of the early embryo [11]. During brain development, the transcriptional activity of En controls the midbrain/hindbrain border [12], participates to the specification and survival of mesencephalic monoaminergic cells [13–15], and regulates cerebellar patterning and connectivity [16,17]. Abnormal cerebellar development, cognitive impairment, and E/I imbalance are observed in *En2* KO mice and are relevant to ASDs [18,19], suggesting that disturbance of En2 expression is functionally linked to ASDs. The En proteins together with the homeoproteins Otx2 and Pax6 also act as extracellular signaling molecules and activate mRNA translation [10]. By this means, En fine tunes the retinocollicular connectivity in the late embryonic period [20,21], and increases the survival of mesencephalic dopaminergic neurons in the adult brain [22]. Most of the assumptions about the role of En2 in autism have been discussed in relation with midbrain and posterior brain where it is highly expressed. However, other studies indicated a role for En in adult limbic structures [18, 23–25].

Recent studies [23–25] and data from brain atlas (http://www.gensat.org) indicate that En proteins are expressed at modest levels in the adult and juvenile hippocampus. We reasoned that any effect of excessive En2 concentration (i.e. as in ASD) may be simpler to characterize in a structure known to express low levels of Engrailed protein, such as the hippocampus. After having re-examined the levels of expression of endogenous En1 and En2 by RT-qPCR, we addressed the effect of an increase in En2 concentration, and by comparison with that of En1, on dendritogenesis, spinogenesis and synaptogenesis in hippocampal neurons. Overall our results reveal a previously unknown ability of En1 and En2 to influence the morphology and the connectivity of forebrain neurons and extend our understanding of how En2 functions may relate to ASDs.

## Materials and methods

### Animals

Mice were treated in accordance with guidelines for the care and use of laboratory animals (US National Institutes of Health) and the European Directive number 86/609 (EEC Council for Animal protection in Experimental Research and Other Scientific Utilization). The experiments were approved by Paris Descartes University ethics committee (Permit Number: CEEA34.FD.047.11.). Mice were euthanized by cervical dislocation and embryos by decapitation.

### Statistical analysis

Data are depicted as mean ± SEM and were analyzed by GraphPad Prism7 software (San Diego, CA). Statistical significance of the differences was calculated by using p<0.05 as the minimum level of significance and one-way ANOVA (or a Kruskall Wallis test if data were not normally distributed, as assessed by D’Agostino and Pearson normality test) followed, if overall p< 0.05, by a multiple group comparison post hoc test or by two-group comparisons with a Student t-test or alternatively a Mann Whitney U test, as mentioned in figure legends.

### Primary hippocampal and glial cell cultures

Primary hippocampal cell cultures were prepared from E16 embryonic Swiss mice (Janvier Labs) as previously described [26]. Briefly, hippocampi were dissected out in pre-chilled (4°C) Neurobasal medium (Gibco) using a stereomicroscope, and the meninges carefully removed. Hippocampi from E16.5 embryos were collected, washed twice with pre-chilled HBSS (Gibco), and incubated for 10–12 min in 2.5% trypsin in EDTA with 2.5% DNAse diluted in HBSS in a 37°C water-bath. The cells were then dissociated by trituration, and plated at a density of 2 500 cells/cm^2^ to 35 000 cells/cm^2^ on poly-D-lysine-coated petri dishes (Falcon, Thermo Fischer Scientific), or glass coverslips when used for immunofluorescence. Plating culture medium consisted of Neurobasal medium supplemented with 2% B27 (Life Technologies, Gaithersburg, MD), GlutaMAX^TM^-I (Invitrogen), and 0.1% penicillin-streptomycin (Gibco). Cultures were maintained at 37°C in a humidified 5% CO_2_ atmosphere. After 2 to 24 hours in vitro, plating medium was replaced by glial cell-conditioned medium.

Primary cultures of glial cells were prepared from PND4 Sprague-Dawley rats (Janvier Labs). Cortices were dissected out and processed as previously described [26]. Cells were plated in 75 cm^2^ flasks in Dulbecco's Modified Eagle Medium with 4.5 g/L glucose and glutamine (DMEM High glucose GlutaMAX^TM^-I, Invitrogen) supplemented with 10% fetal calf serum (FCS) and 0.1% penicillin-streptomycin (Gibco). Cells were let to grow at 37°C in a humidified 5% CO_2_ atmosphere until they were sub-confluent (80%) and passaged to 1:2 in same medium. Passages #1 and #2 were used to prepare conditioned medium as followed: DMEM medium was replaced by pre-warmed Neurobasal medium supplemented with 2% B27, GlutaMAX^TM^-I and 0.1% penicillin-streptomycin for 24h to 48h, then filtered and used to feed primary hippocampal neurons. The conditioned medium was not replaced over the time of the hippocampal culture.

### Transfections

Cells were transfected with Lipofectamin2000® (Invitrogen) between 10 and 12 days in vitro (DIV). Neuron growth medium was kept away except 100 μL per well to which was added Lipofectamin2000® diluted in 100 μL Neurobasal medium at 2.5% v/v and 2.5 μg plasmid expressing soluble enhanced GFP (EGFP). Cells were incubated at 37°C, 5% CO_2_ for 3h; transfection medium was then replaced by neuron growth medium up to 1000 μL.

### RT-qPCR

**Fig 1:**
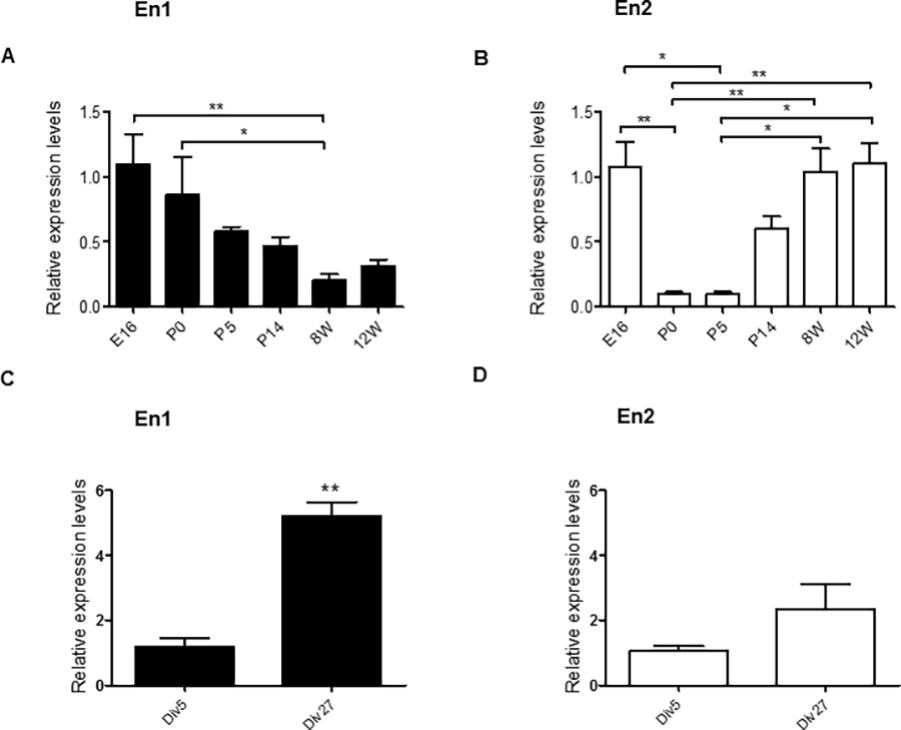
Expression of Engrailed 1 and 2 in the hippocampus. (A-D) RT-qPCR using RNAs extracted from the hippocampus at various embryonic (E) and postnatal days/weeks (P, W) (A, B), or from hippocampal cells at different day in vitro (Div) (C, D). Primers used (primers #1, see Material & Methods) are specific for En1 (A, C) or for En2 (B, D). Normalized values are the mean +/- s.e.m. from a minimum of n = 4 independent experiments for each developmental age and time. Significance of the differences were assessed with a Kruskal-Wallis test, in A (***, p = 0.0006) and B (****, p<0.0001), followed by a Dunn's Multiple Comparison Test. *, p<0.05; **, p<0.01. Detailed P-values are given in supplementary information (S1 Appendix).

Total RNAs were extracted from primary cell culture of hippocampal neurons or alternatively from isolated hippocampal tissues with the DNA/RNA/Protein minikit (Qiagen). 1μg of RNA was reverse transcribed with GoScript Reverse Transcription System (Promega) according to the manufacturer instructions. Quantitative PCR was performing using ABI Prism 7900HT device (Applied Biosystem) with real-time detection of fluorescence. Q-PCR reactions were conducted in triplicate in a volume of 10 μL using QuantiTect ® SYBR® Green PCR Kit (Qiagen) according to manufacturer instructions. Mouse hypoxanthine Guanine Phosphorybosyltransferase (HPRT) was used as standard for quantification. Primers #1 (MWG biothech) were as follows: HPRT forward, AGCAGGTGTTCTAGTCCTGTGG; HPRT reverse, ACGCAGCAACTGACATTTCTAA; En1 forward, GTGGTCAAGACTGACTCACGC; En1 reverse, GCTTGTCTTCCTTCTCGTTCTT; EN2 forward, ATGGGACATTGGACACTTCTTC: and EN2 reverse, CCCACAGACCAAATAGGAGCTA. Another set (primers #2) of previously validated primers for En1 and En2 [27] were also used in this study (S1 Fig). 40 PCR cycles (15” at 95°C, 30” at 55°C, and 30” at 72°C) were carried out. Dissociation curves were consistent with a single product being amplified with the different couples of primers. To compute relative expression levels (2^ΔΔCt), we first subtracted the mean Ct of GAPDH or HPRT to Ct values of EN1 or EN2 measured in the same samples to get DCt; from these values, we then subtracted DCt measured at E16 in the hippocampus to get ΔΔCt. Data in Fig 1 and S1 Fig are thus shown as fold changes relative to the expression of EN1 or EN2 at E16 in the hippocampus.

### Antibodies and immunofluorescence

Neurons were fixed with pure methanol pre-chilled at -20°C for 5min, then rinsed in PBS. Permeabilization was performed with 0.1% Triton-X100 in PBS and cells were incubated in blocking solution (5% FCS in PBS) 30 min at room temperature. For phospho-S6 labelling, neurons were fixed in 4% paraformaldehyde and 4% sucrose in PBS, permeabilized with 3% Triton-X100 in PBS for 3min, incubated in blocking solution (3% BSA in PBS) for 30min and then with the anti-Phospho-S6 antibody in blocking solution for 1h. The following antibodies were used: anti-mouse PSD95 (1:500, Thermo Fischer), anti-rabbit VGlut1 (1:300, a kind gift from Dr. El Mestikawy, Paris, France), anti-mouse Map2B (1:1000, Millipore), anti-chicken Map2B (1:500, Clinisciences), anti-glutamic acid decarboxylase (GAD67) (1:2000, Synaptic Systems), rabbit anti-Phospho-S6 ribosomal protein (Ser240/244, 1:800, Cell signaling), anti-VIAAT (1:100, a gift from Dr. B. Gasnier). Primary antibodies were diluted in 1% FCS in PBS and incubated overnight at 4°C. After several rinses in PBS with 1% FCS, coverslips were incubated with the appropriate Alexa-conjugated secondary antibodies (Invitrogen, Carlsbad, CA) diluted in PBS plus 1% FCS and 0.01 μg/mL Hoechst 33342 for 45 min at RT, rinsed extensively in PBS, and mounted with Fluoromount® anti-fading media.

### Hippocampal cell treatments

Hippocampal cells were treated daily with recombinant En1 or En2 (at the final concentration of 150ng/ml), either for 6 days (i.e. DIV4 to DIV9) for dendritic tree, or 4 days (i.e. DIV15 to DIV18) for dendritic spine analysis.

### Image acquisition and analysis

#### Epifluorescence imaging and dendritic complexity analysis

Neurons and dendritic branches were imaged using a Nikon TE2000-E equipped with a CCD camera (CoolSNAP HQ, Photometrics®) with 40x (NA = 1.3) and 100x (NA = 1.4) objectives. Acquisitions were made with MetaVue^TM^ (Imaging Research Software). Automatic (see supplementary methods) or manual tracing of dendrites of hippocampal cells immunolabelled for Map2B and GAD67 were realized and images were analyzed with ImageJ 1.44o software.

#### Confocal microscope imaging and dendritic spines analysis

GFP-labeled neurons were imaged using a Zeiss Axio Imager M2 microscope with an objective 63/1.50 with adequate laser intensity and signal detection. Images were acquired using ZEN 2011 software. Serial optical sections of 138 nm were taken through the cells. About fifteen neurons per experimental group were captured per experiment, and data from two-three independent experiments were pooled for analysis (an average of 140 μm of dendritic length per neuron was analyzed). In each analyzed condition, only spines of primary and secondary dendrites were counted taking care to consider equal proportions of primary and secondary branches and to avoid the broad dendritic trunk immediately emerging from the cell body. Z-stacks were delivered to Imaris software (Bitplane Scientific Software, 7.0); dendrites and dendritic spines were reconstructed and analyzed using the Filament Tracer module using "autopath" mode; minimum dendrite diameter was set at à 0.25 μm. Spines (minimum spine diameter set at 0.1 μm and maximum length at 3 μm, [28]) were defined as protrusions that could be differentiated from the dendritic shaft and restricted to those that were visible in the x- and y-axes. Dendritic spine density and number of branched spines were automatically calculated. Dendritic spines were classified into "stubby", "mushroom" or "thin" via the "Spine classifier" MathLab module integreted into Filament Tracer with the following settings: for stubby, "length(spine) < 1 AND 0.9 < min_width(spine) / mean_width(head) < 1.1"; for mushroom, "length(spine)<3 AND mean_width(head) / max_width(neck) > 0.75"; for thin, "mean_width(head) / length(spine) < 0.5". The good fit of spine parameters was controlled on a subset of dendritic spines visually identified and manually measured with Imaris Measurements module. All spine measurements were made on z-stack images of dendritic segments.

#### Confocal microscope imaging- PSD95-vGlut IF analysis

Image acquisition was performed using an Olympus IX81 inverted microscope equipped with a FV1000 laser scanning unit with a 40 x objective (oil immersion, NA = 1.30). Two lasers were used, a blue laser diode (473 nm) for the vGlut green labeling, and a green HeNe gas laser (543 nm) for the red labeling of PSD95. Stacks of images were acquired with a sampling rate of 70 nm/pixel (2D spatial resolution, 130nm, after image deconvolution), and a z step of 0.5 μm. Image processing and analysis were executed using the ImageJ 1.47m software. Residual blurring was removed by spatial deconvolution: point spread functions (PSF) were calculated using the ImageJ’s plugin PSF Generator [29] and the deconvolution was performed using the Richardson-Lucy algorithm implemented into the DeconvolutionLab ImageJ’s plugin [30]. These two freeware programs were provided by the Biomedical Imaging Group of the EPFL (Ecole Polytechnique Fédérale de Lausanne; Switzerland). To simplify the analysis, images representing the maximum projection of z-stacks were used for further analysis with an original ImageJ program shortly described below. Briefly, maximum projections were deblurred by a custom unsharp mask filter and denoised by removing outlier pixels. Objects of interest were detected by an optimized thresholding method. Smallest objects (one pixel) were removed from images by the denoising. Further subtraction of potential background was completed by a sorting procedure based on the signal/noise ratio measurement (see Supplementary Information) which eliminated most of the objects with apparent diameters less than 0.335 μm (i.e. corresponding to real diameters of about 0.2 μm). To summarize, most of objects whose size was less than to 0.2 μm were considered as background and excluded from the analysis. VGlut and PSD95 detected objects were then submitted to an overlapping analysis into user-defined selections delimiting the dendritic edges. Red and green objects were considered as synaptic elements when their apparent area overlapped more than 60 pixels (about 0.25 μm^2^) (see Supplementary information). Object density was calculated by dividing the number of object per measured selected dendrite areas.

### Golgi-Cox® staining

En2 KO mice [18,23], mice heterozygous for En1 (provided by Dr Alain Prochiantz, Collège de France, Paris, France) and WT mice were sacrificed, their brains were quickly removed and processed for Golgi staining as indicated by manufacturer (Rapid GolgiStain^TM^ FD Neurotechnologies). 200 μm-thick brain sections were realized using a vibratome (Leica Microsystems), then were mounted on histological slides and revealed as indicated by manufacturer. Images from basal and apical dendrites of CA1 pyramidal cells, and from dendrites of granular cells in the dentate gyrus were realized using a Nikon microscope with a 60X/1.40 objective. Dendritic spines were counted manually using ImageJ software, spine density was calculated by dividing the number of spines per measured dendrite length.

### SUnSET

SUnSET, for surface sensing of translation, was adapted from [31]. SUnSET is based on the incorporation of puromycin, an analog of tyrosyl-tRNA, into nascent peptide chains, leading to the termination of elongation and the accumulation of puromycin-conjugated peptides which can be detected by immunofluorescence. Neurons were incubated at 37°C, 10min with 2μg/ml puromycin (Sigma) in conditioned medium (CM) followed by rapid neurobasal washes and 15 min chase in CM. After paraformaldehyde fixation, triton X-100 permeabilization and blocking, puromycin was detected with a monoclonal anti-puromycin antibody (12D10, 1:800, Millipore corporation) and Alexa Fluor 555 goat anti-rabbit IgG or Alexa Fluor 488 donkey anti-mouse IgG (Invitrogen). Cells were co-stained with an anti-MAP2 antibody. Images were taken using a z-motorized Nikon inverted microscope TE2000E and analyzed with ImageJ software (http://imagej.nih.gov/ij/). Shown is the mean fluorescence, which was obtained by dividing the integrated fluorescence intensity of each image by the cellular surface area determined by automatic thresholding (using the “triangle” macro in ImageJ) of the MAP2 image.

## Results

### Profiles of En1 and En2 expression in the developing hippocampus

The first aim of this study was to re-examine the expression of En mRNA in hippocampal cells. Low but significant levels of En1 and En2 mRNAs were detected by RT-qPCR in mouse hippocampi and cultures of mouse hippocampal neurons. (Fig 1, S1 Fig). Similar temporal patterns of expression were observed in different mice with two different couples of primers, including primers that have been validated in Engrailed KO mice [27], arguing against a possible lack of specificity of these probes. The expression of En1 and En2 in the hippocampus were found to span periods of time corresponding to neurogenesis (E16-P0), synaptogenesis (P0-P14), and synaptic maturation (P14-3 months) [32,33]. En1 and En2 showed distinct, albeit complementary, expression patterns during these periods. En1 expression levels were highest between E16 and P5 and then decreased to reach in young mice (12 weeks) less than half of the levels they had at E16 (Fig 1A, S1 Fig). In sharp contrast, the expression of En2 abruptly fell between E16 and P5, and then resumed (Fig 1B, S1 Fig). In hippocampal cell cultures, En1 expression increase by about 5 times between the low differentiated neuronal stage (i.e. DIV5) and the highly differentiated hippocampal network stage (i.e. DIV27), whereas En2 expression level remained weak during the same period (Fig 1C and 1D). Thus, the expression of En2 seems to be tightly controlled when synaptogenesis is activated (first postnatal week and late cell culture stages), and when En1 appeared as the major En isoform.

### An excess of En affects dendritic morphology and spinogenesis

**Fig 2:**
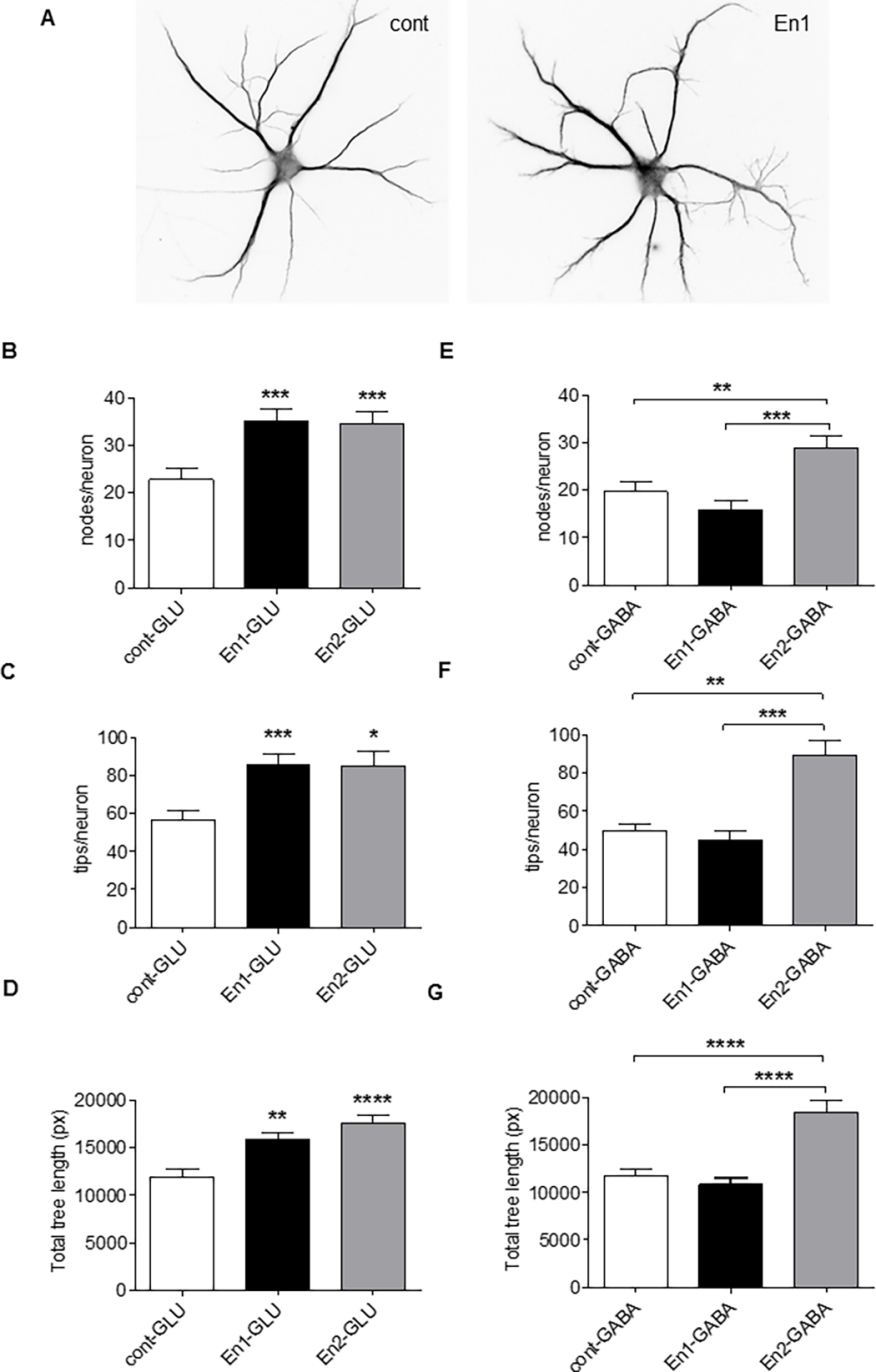
Exogenous Engrailed increases dendritic complexity. (A) Div9 hippocampal cells not-treated (A, left) or daily treated with 150ng/ml of purified En for 6 days (div4-div9, A, right) and immunolabelled with MAP-2. Gabaergic cells were identified by immunolabelling with an anti-GAD67 antibody. (B-G) quantification of dendritic nodes (B, E) dendritic tips (C, F), and dendritic length (D, G) per non-treated pyramidal cells (cont) and pyramidal cells treated with either En1 or En2 (B-D), and per non-treated GABA cells (cont) and GABA cells treated with either En1 or En2 (E-G). Values are the mean +/- s.e.m. of measures from a total of 40–45 pyramidal cells and from a total of 30–40 GABA cells/condition from 3 (En1) and 2 (En2) independent experiments. Significance of the differences were assessed with a Kruskal-Wallis test, in B, D, F, G (****, p< 0.0001), C (***, p = 0.0006), and in E (***, p = 0.0002), followed by a Dunn's Multiple Comparison Test. *, p<0.05; **, p<0.01; ***, p<0.001; ****, p<0.0001. Detailed P-values are given in supplementary information. Bar in A, 35μm.

The elevated En2 gene expression in the brain of individuals with autism [8, 9] prompted us to investigate the effects of an excess of En2 and, by comparison, of an excess of En1, on hippocampal cell morphology. Extracellular En proteins guide and stabilize axons both in Chicken and Xenopus [20, 21, 34, 35]. Building on these previous studies and on the capability of En and other homeoproteins to act as signaling molecules [10], we added the recombinant protein in the cell culture medium. We found that daily treatment with either En1 or En2 (from DIV4 to DIV9) of young isolated neurons (Fig 2A), increased the dendritic complexity of glutamatergic neurons as revealed by an increase in the number of nodes (Fig 2B), of dendritic tips (Fig 2C) and of total dendritic length (Fig 2D). Strikingly, the complexity of GABAergic dendritic tree was also increased by En2, but was insensitive to En1 (Fig 2E–2G), indicating a broader function for the En2 isoform. These effects appear to depend on Engrailed proteins internalization as dendritic complexity was essentially unchanged after treatments with mutants (En1SR and En2SR [34]) that do not have the capacity to cross the cell membrane (S2 Fig).

**Fig 3:**
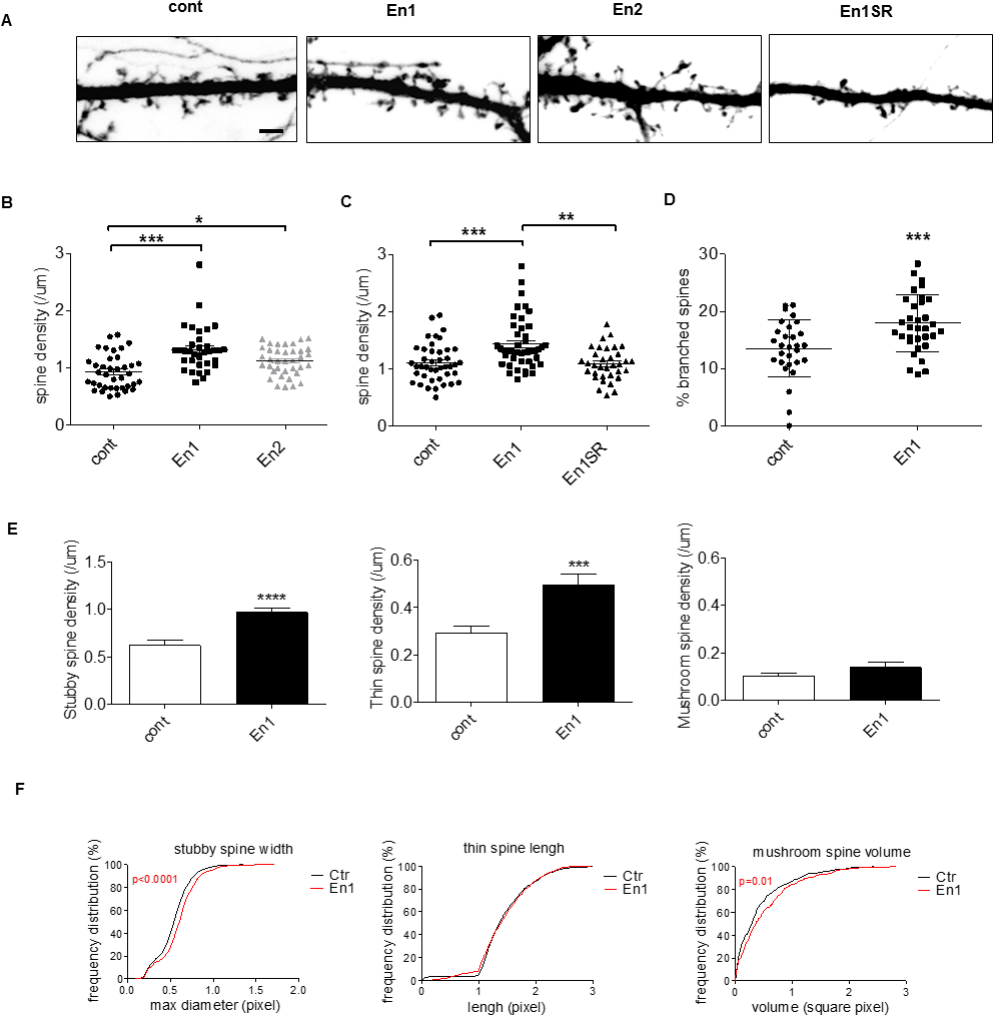
Engrailed increases immature spine density in vitro. (A-E) Hippocampal cells were transfected with pEGFP, daily treated with 150ng/ml of Engrailed or En1SR, a membrane non-permeant mutated construct, from div15 to div18 and imaged at div19. (B) Quantification of dendritic spine density after treatment with either En1 or En2, or (C) after treatment with either En1 or En1SR. Significance of the differences were assessed with a Kruskal-Wallis test in B (****, p< 0.0001), and C (***, p = 0.0001), followed by a Dunn's Multiple Comparison Test. *, p<0.05; **, p<0.01; ***, p<0.001. Values are the mean +/- s.e.m. of measures from 3 independent experiments with an average of 39 pyramidal cells per condition. (D) Quantification of branched spines after treatment with En1. ***, p = 0.0004 (t-test). (E,F) Morphometric analysis of dendritic spines under control conditions or after En1 treatments. (E) Spines were categorized using Imaris software as described in Methods. En1 increased the density of “stubby”, ****, p<0.0001 and “thin” spines, ***, p = 0.0002 (t-test) but not the one of “mushroom” spines, p = 0.1874 (Mann-Whitney test). (F) En1 increases the maximum diameter of stubby spines (KS test, D = 0.159, p<0.0001), does not increase thin spine length (KS test, D = 0.06, p = 0.054), and slightly increases mushroom spine volume (KS test, D = 0.119, p = 0.01). Values in D-F are the mean +/- s.e.m. of measures from 2 independent experiments each cumulating an average of 30 pyramidal cells per condition. Bar in A, 5μm.

Next, we determined the effect of a daily En treatment of mature neurons which have formed a dense network (i.e. from DIV15 to DIV19). As illustrated in Fig 3A–3C, we found that En1 treatment of mature glutamatergic neurons for four days increased dendritic spine density by 32% compared to control, while treatment with En2 did increase it by 18% (Fig 3B). The increase in spine density also required the internalization of En because En1SR had no effect (Fig 3C). We next categorized dendritic spines in branched, mushroom, stubby and thin spines according to their shape and asked which types of spines were increased upon treatment with En1 (Fig 3D and 3E). Compared to control, En1 increased the number of branched spines (Fig 3D), and the density of stubby and thin spines (Fig 3E). A similar, but non-significant, trend was observed on mushroom spines (Fig 3E). Morphometric analysis additionally indicated that En1 significantly enlarged stubby spines and only slightly increased mushroom spine volume, while it did not affect at all the length of thin spines (Fig 3F). Altogether these results suggest that increasing En1 concentrations during a period of synaptic differentiation and maturation induced the formation or the stabilization of immature (stubby) and of plastic spines (thin, branched).

Having established a link between En and spinogenesis *in vitro*, we addressed this issue in *in vivo* models. Mice overexpressing En in the hippocampus are not available yet, but *En1* and *En2* knockout mouse lines have been established. As *En1*^-/-^ mice are not viable, we measured spine density in *En1*^+/-^ and *En2*^-/-^ mice by staining their hippocampi with the Golgi method (S3 Fig). We found that the density of dendritic spines was changed in these mice but according to a regional pattern (S3 Fig). In adult *En1*^+/-^ mice, the density of spines was decreased on CA1 apical dendrites but unchanged on CA1 basal dendrites and in DG, compared with age-matched control littermate mice (S3 Fig). By comparison *En2* KO displayed no significant changes in spine density on apical and DG dendrites, and a slight increase on basal dendrites (S3 Fig). These observations demonstrate that En1 is important *in vivo* to control spine formation or stabilization.

### Increase of En concentration affects the synaptic density within hippocampal cells

**Fig 4:**
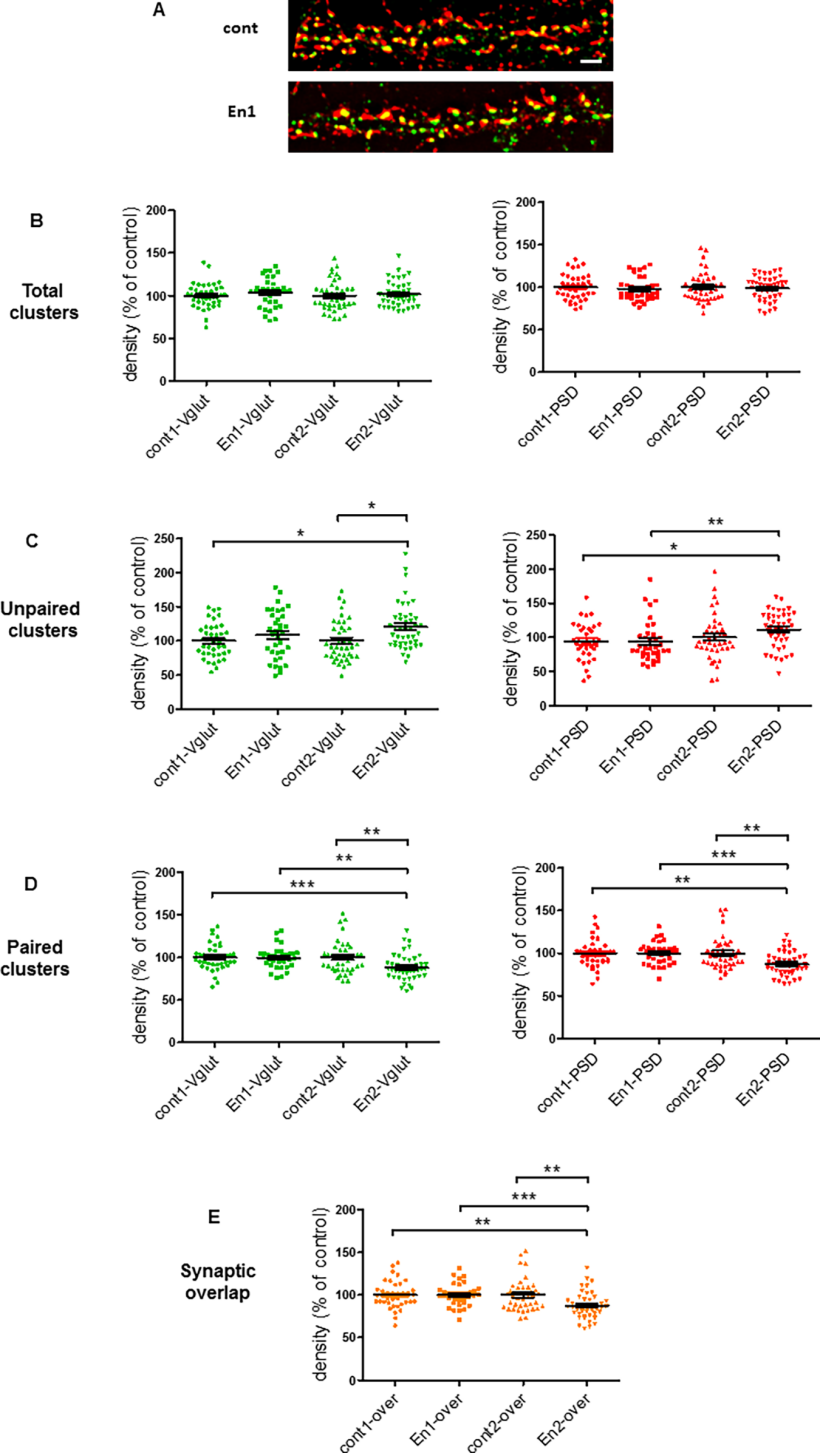
En2 reduces synaptic pairing. (A) Confocal images of hippocampal cell dendrites double stained for PSD95 and vGlut1 without treatment (cont) or after daily treatment (div15-div18) of hippocampal cells with 150ng/ml of either En1 or En2. (B-E) Quantification of vGlut1 and PSD95 cluster densities (in green and red, respectively) in cells treated as in A (per μm^2^). Shown are analysis for total clusters (B), single clusters (i.e. vGlut1 and PSD95 not paired with each other, C), paired clusters (i.e. vGlut1 and PSD95 puncta whose area overlapped more than 0.25 μm^2^ as defined in S1 Appendix, D) and overlap (yellow clusters, i.e. immunofluorescent overlap resulting from pairing of vGlut1 and PSD95, shown in brown in E). Values are the mean +/- s.e.m. of 44 measures from 3 independent experiments. Kruskal-Wallis test in C (**, p = 0.0099), and D, E (***, p = 0.0001), followed by a Dunn's Multiple Comparison Test. *, p<0.05; **, p<0.01; ***, p<0.001. Detailed P-values are given in supplementary information. Bar in A, 5μm.

Since En affected spinogenesis, we wondered whether the homeoproteins also had an impact on synaptogenesis. To analyze synaptogenesis in cell cultures we labelled cultured hippocampal neurons with antibodies recognizing PSD95 and the vesicular glutamate transporter vGlut1, two standard markers of postsynaptic and presynaptic compartments, respectively (Fig 4A). Confocal images were processed with locally-developed routines which allowed accurate segmentation of pre- and post-synaptic structures as well as the synaptic contacts (see Materials and Methods, S5 Fig and S1 Appendix). In contrast to their effect on spine density, En proteins did not increase the density of total PSD95 clusters (Fig 4A red puncta and 4B). En did not change either the total density of presynaptic clusters (Fig 4A green puncta and 4B). To further analyze the effect of En on synapses, we evaluated the pairing of synaptic partners. For this, we quantified the density of PSD95 or vGlut1 puncta that do not form a synapse (i.e. single red or single green puncta, respectively) (Fig 4C). We also quantified the density of puncta forming a synapse, either by measuring the density of red and green puncta whose area overlapped more than 0.25 μm^2^ (i.e. paired puncta, Fig 4D), or the density of yellow puncta corresponding to the immunofluorescent overlap between red and green puncta (Fig 4E). These quantifications revealed that En2, but not En1, increased the number of single unpaired PSD95- and vGlut1-IR puncta, and correlatively reduced the number of paired and overlapped PSD95/vGlut1 puncta.

**Fig 5:**
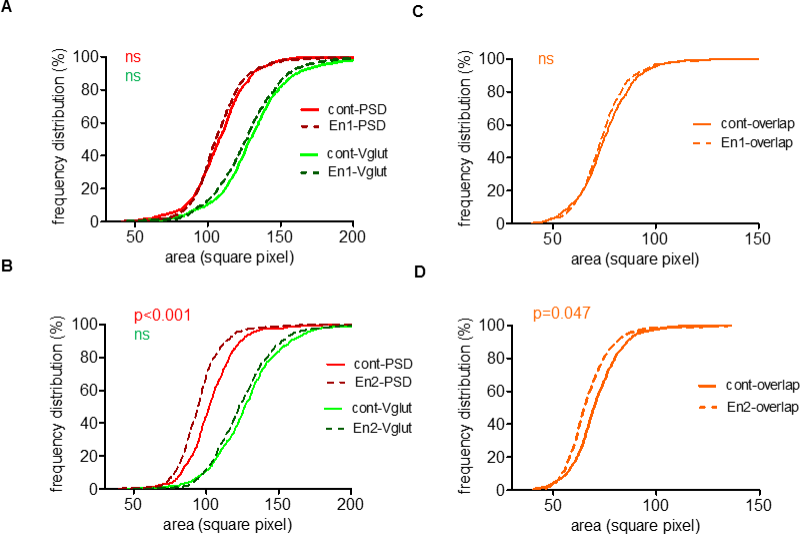
Engrailed-2 reduces synaptic cluster area. (A-D) Frequency distribution of the area of vGlut1 (green) and PSD95 (red) (A, B) and vGlut1/PSD95 overlap (i.e. synaptic overlap, brown) (C, D) clusters analyzed in Fig 4. Kolmogorov-Smirnov test on two samples (http://www.physics.csbsju.edu/stats/KS-test.html): A, cont-PSD vs En1-PSD (D = 0.072, p = 0.178), cont-Vglut vs En1-Vglut (D = 0.084, p = 0.055); B, cont-PSD vs En2-PSD (D = 0.183, ***p<0.0001), cont-Vglut vs En2-Vglut (D = 0.055, p = 0.568); C, cont-overlap vs En1-overlap (D = 0.074, p = 0.208); D, cont-overlap vs En2-overlap, (D = 0.112, *, p = 0.047).

In addition, En2, but not En1, lowered the postsynaptic local concentration of PSD95 as indicated by the reduction of the area of PSD95 clusters (Fig 5A and 5B). Consistently, En2 specifically lowered the area of synaptic contacts (Fig 5C and 5D). However, none of the two proteins had an effect on the area of presynaptic vGlut clusters (Fig 5A and 5B). Collectively, our results indicated that excessive concentration of En2 specifically altered glutamatergic synaptic pairing on dendrites. Looking for an effect of En treatment on GABAergic connectivity, we found that En proteins slightly increase the density of VIAAT-positive presynaptic contacts on glutamatergic cells (S4 Fig).

### An excess of Engrailed increases protein synthesis in hippocampal cells

**Fig 6:**
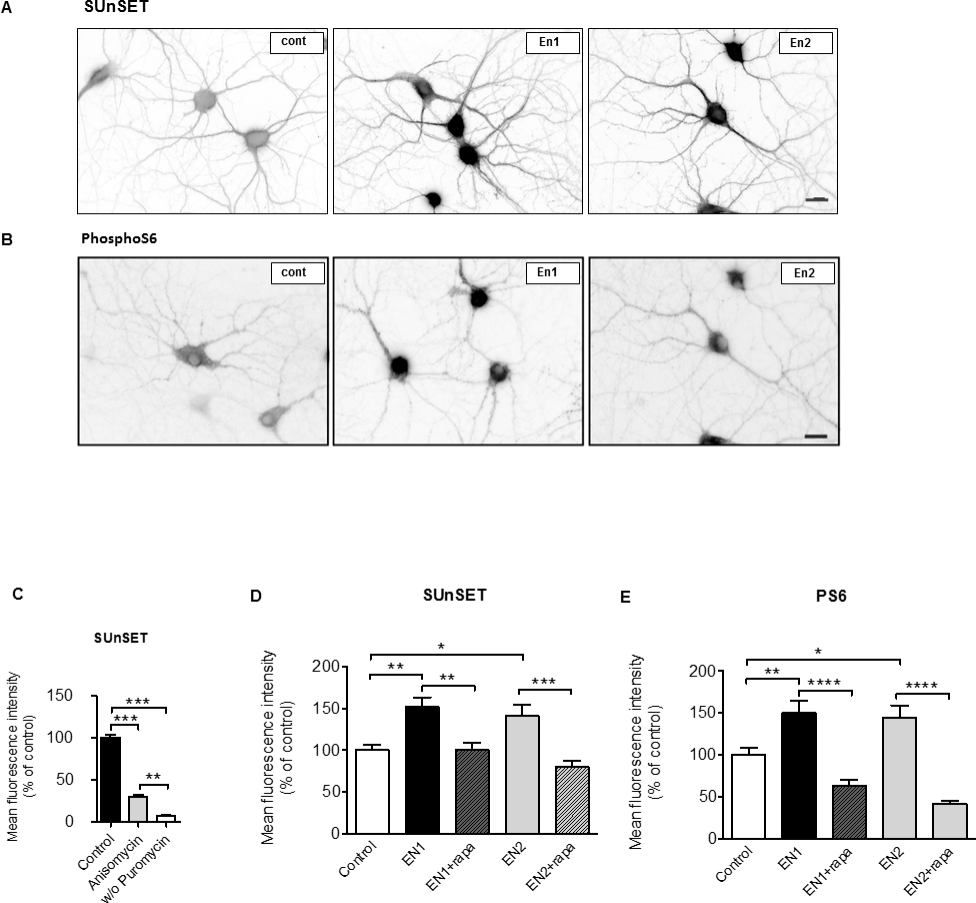
Engrailed increases mTORC1 activity and protein synthesis in hippocampal cells. **(**A) De novo protein synthesis in hippocampal neurons visualized with SUnSET. DIV18-20 neurons were analyzed without treatment (cont) or after a 1-hr treatment with either En1 or En2. Shown are inverted grayscale images. (B) Phosphorylation of S6 (Ser240/244) assayed by immunofluorescence in untreated hippocampal neurons (cont) or in neurons treated as in A. (C) The SunSET signal was strictly dependent on the presence of puromycin and strongly suppressed by anisomycin indicating that it was reporting de novo protein synthesis. (D) Quantification of SUnSET images. (E) Quantification of phospho-S6 images. Values in D and E are the mean (+/- s.e.m.) fluorescence intensity in neurons from N = 19–22 images from 2 independent experiments. D and E, One-way ANOVA (****, p< 0.0001) followed by a Tukey’s Multiple Comparison Test. C, Kruskal-Wallis test (**** p< 0.0001) followed by Dunn's Multiple Comparison Test. *, p<0.05; **, p<0.01; ***, p<0.001; ****, p<0.0001. Detailed P-values are given in supplementary information. Bar in A and B, 20μm.

Compelling evidence suggests a link between exaggerated protein synthesis and ASD [5, 36], and autism-related deficits have been recently associated to dysregulation of eIF4E-dependent translational control [37, 38]. Since En induces eIF4E phosphorylation and activates local protein translation in retinal growth cones [20, 34], we wondered if the concentration of Engrailed used here (4nM) could also increase protein synthesis. To test this possibility, we used SUnSET, a method based on the incorporation of puromycin into newly synthesized proteins followed by immunodetection of puromycylated polypeptides [31]. As expected, the SUnSET signal depends on the addition of puromycin and is strongly reduced by anisomycin, an inhibitor of protein synthesis (see Fig 6C). Using this assay in hippocampal cells, we found that protein synthesis was increased by about 50% with En1, and 41% with En2, relative to baseline conditions (Fig 6A and 6D). The mechanistic target of rapamycin complex 1 (mTORC1) is a key regulator of mRNA translation [39]. We addressed the impact of En1 and En2 on mTORC1 by measuring the phosphorylation of the ribosomal protein S6. Both En1 and En2 increased the amount of phospho-S6 (Fig 6B and 6E) and this effect is suppressed by rapamycin, an inhibitor of mTORC1. The data suggest that the effect of En1 and En2 on dendrites and dendritic spines may be mediated by an increase in mTORC1-dependent protein synthesis.

**Fig 7:**
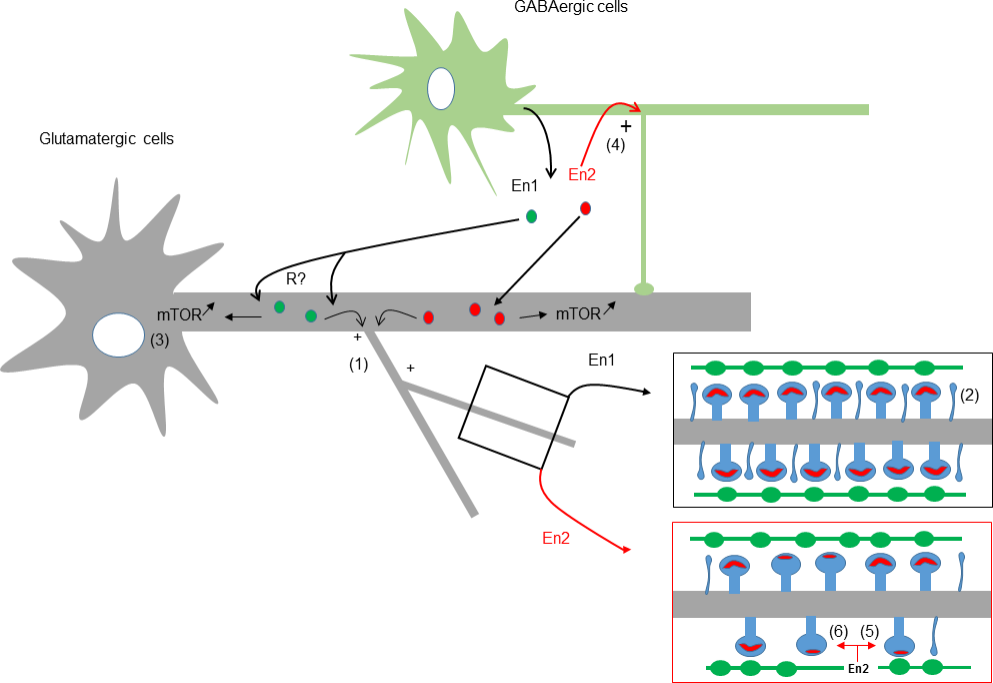
Modelling of the effects of En1 and En2 on hippocampal cells. Coarse hypothetical scheme of the Engrailed signaling pathway in the normal and autistic brain. Engrailed proteins are known to be mainly expressed by GABA neuronal cells [23], and to signal between cells upon secretion (reviewed in [10]). In normal conditions (black lines), low levels of endogenous secreted En1 (from GABA cell for instance) could participate to dendritogenesis (1), spinogenesis (2) (insert), and mTOR signaling (3) in neighbor glutamatergic neurons. In a pathological context, such as autism (red lines), a rise of En2 expression and secretion would generate additional effects, i.e. the increase in dendritic complexity of GABA cells (4), a decrease of clustered PSD95 (5), and the mispairing of glutamatergic synapses (6) (insert). Engrailed proteins require cellular internalization to be active, but functional priming at the cell membrane through an uncharacterized protein “R” is not excluded.

In conclusion, our results indicate that increasing the concentration of En1 and En2 produce similar but also different effects summarized in Table 1. Particularly interesting in the context of autism were the observations that En2, but not En1, can increase GABA cells complexity, and reduce the density of glutamatergic synapses. Based on the present results and the known expression of En proteins in hippocampal interneurons [23], we established a sketch modeling the predictable effects of unsuitable levels of secreted En proteins on hippocampal network (Fig 7).

**Table 1:** Summary of the effects of En1 and En2 on hippocampal cells.

Comparative caracteristics of En1 and En2		En1	En2	En1SR	En2SR
Expression level during synaptogenesis in hippocampus (qPCR)		low	weak		
Effect on:					
	complexity of glutamatergic dendrite	increase	increase	low increase	ns
	complexity of GABAergic dendrite	ns	**increase**		ns
	density of dendritic spines	increase	increase	ns	
	spine type density				
	stubby	increase			
	thin	increase			
	mushroom	ns			
	branched	increase			
	Synaptic clusters				
	presynaptic (Vglut) density	ns	ns		
	postsynaptic (PSD95) density	ns	ns		
	synapses (Vglut/PSD95 overlap) density	ns	**decrease**		
	presynaptic (Vglut) cluster area	ns	**decrease**		
	postsynaptic (PSD95)cluster area	ns	**decrease**		
	presynaptic (VIIAT) density	low increase	ns	ns	
	Protein synthesis				
	PS6 signal	increase	increase		
	SUnSET signal	increase	increase		

Table summarizing the different effects of En1 and En2 reported in the present work. Shaded boxes: non-determined; in bold characters: effects specific for En2; ns: not significantly different from control.

## Discussion

We found here that excessive signaling by En alters dendritogenesis and spinogenesis and thus the accurate development of hippocampal cell network. It is well established that En drives important aspects of the development of midbrain and cerebellum [12,16]. However, the possibility of a role of En in the forebrain, which could better reflect the cognitive impairments observed in ASDs and *En2*^-/-^ mice [1,25], has been disregarded, possibly because of the highest expression level of En in the midbrain/hindbrain regions (http://www.gensat.org). We revisited the expression of En in the hippocampus, confirmed precedent reports showing that En are expressed in the adult hippocampus and hippocampal cells [18,23–25], and further showed that the expression of En1 and En2 is subjected to a tight regulation during the development of this structure. Particularly striking is the downregulation of En2 mRNA during the first postnatal week, a period associated with a sudden increase in synaptogenesis in rodents [33]. A similar downregulation of En2 at birth has been reported in the cerebellum [40]. This downregulation is functionally important as maintenance of En2 overexpression postnatally in Purkinje cells delays their differentiation [41], a phenomenon hypothesized to occur during ASD [42]. Considering the ability of En2 to reduce excitatory synaptic contacts, the sharp downregulation of En2 mRNA at P0-P5 may usefully prevent interference between En2 effects and glutamatergic synaptogenesis. Differential expression of En1 and En2 during hippocampal development suggests that these two genes have distinct activities in relation to neuronal differentiation and maturation. Accordingly, we found that En2 increased GABAergic dendritic complexity and reduced glutamatergic connectivity, while En1 did not. On the other hand, En1 was more effective than En2 to increase spinogenesis on pyramidal neurons *in vitro*. This functional partitioning is maintained *in vivo* in the adult hippocampus since En2 KO but not En1+/- mice display a loss of GABAergic neurons [15, 23], while spine density is reduced in the hippocampus of adult En1+/- but not in En2 KO mice (present results). Still, the functional specificity of En proteins does not exclude some redundancy as evidenced by the finding that, *in vitro*, both En1 and En2 promote dendritic complexity and spinogenesis in glutamatergic cells.

The shape of the dendritic tree is a morphological hallmark of neuronal subtypes. Therefore, by acting on glutamatergic and GABAergic dendritic branching, En1 and En2 may influence the formation of neuronal networks. However, given the very weak expression of En2 in the hippocampus at birth, En1 may be the main active isoform at this developmental stage. In line with this hypothesis, spine density was decreased in En1+/- but not in En2 KO. In this study, we addressed what may happen when the concentration of En2 is abnormally increased in the neonate hippocampus. We found that moderately increasing the level of En2 (4nM) was enough to significantly increase GABAergic dendritic complexity, and to reduce the number of excitatory synaptic contacts on glutamatergic neurons, suggesting that these parameters are very sensitive to altered levels of this homeoprotein. These results suggest that an excess of En2 signaling may alter the excitation/inhibition (E/I) balance as observed with several mouse models of ASD “risk genes” [2, 43]. For instance, enhanced GABAergic innervation is found in mice carrying the human R415C mutation in the gene encoding Neuroligin-3 [44], while other ASD models exhibit a loss of GABAergic function [43]. The reduction of PSD95 in En2-treated cells may contribute to changes in the E/I balance. Indeed, an increase in the E/I synaptic current is observed in neurons overexpressing PSD95, whereas knockdown of PSD95 reduces the E/I ratio [45, 46]. Additional studies are needed to determine how the timing of En2 overexpression may functionally affect the hippocampal network and the E/I balance *in vivo*. Noteworthy, previous studies have shown that other members of the homeoprotein family, namely the homeobox proteins Cux1 and Cux2, modulate dendrite branching, spine development and synapse formation in layer II–III neurons of the cerebral cortex [47].

The En-induced spinogenesis observed in this study resulted in an increase of immature spines (thin and stubby) that were not associated to a net increase in the density of PSD95 clusters and synapses. An apparent uncoupling between spines and PSD95 clusters formation could in theory be explained by the integration of the newly formed spines into multi-synaptic contacts, but we detected only few multi-synaptic contacts with no statistical differences among experimental conditions (see S1 Appendix). Alternatively, the newly formed spines may have low amounts of PSD95, or small clusters that remain below the selection threshold (see Material and Methods and S1 Appendix). This is in indeed conceivable as our imaging processing did not allow to quantify the spines having the smallest PSD95 cluster area (i.e. with an apparent area less or equal to 0.1 μm^2^ or a real area below 0.04 μm^2^, [48]) and because low amounts of PSD95 are often correlated to less differentiated spines (i.e. stubby and thin; [49–52]), precisely those enriched by En1. Immature and plastic thin or stubby spines are thought to represent the substrate of both experience-dependent wiring and rewiring of neuronal circuits [53,54]. Therefore, by favoring the increase of stubby and thin spines over the synaptogenesis period (i.e. two first postnatal weeks in mice) En may influence, either physiologically or pathologically, the mechanisms that fine tune the formation of the hippocampal cell network. Consistent with this idea are our observations that En1+/- mice displayed reduced levels of spines in the hippocampus, and that En increased the number of branched spines in vitro. Branched spines increase when animals are put in an enriched environment [55] or after long-term potentiation (LTP) [56], and have been proposed as a possible structural substrate of synaptic plasticity.

Protein synthesis is crucial for long term dendritic and synaptic plasticity, but excessive local mRNA translation may also form the pathogenic core of ASD-linked diseases such as fragile-X syndrome (FXS) and tuberous sclerosis (TSC) [5,57]. In line with the capacity of En to activate mRNA translation in axonal growth cones [20,36] we discovered here that nanomolar concentrations of En1 increase mRNA translation in hippocampal cells. Translational signaling pathways, involving phosphorylation of the elongation factor eIF4E, the PI3K (phosphoinositide 3-kinase)/Akt/mTOR and ERK (extracellular signal-regulated kinase) cascades, are viewed as a common endpoint in various ASD-linked gene mutations and copy number variations (CNVs) [5]. We found here that En1 and En2 increase the activity of mTORC1, an effect that may account for the observed increase in mRNA translation and spinogenesis.

Our data bring new elements to the discussion of the links between En2 and ASDs. First, the increase in spine density observed upon En treatment is reminiscent of the increased spine density found in samples of individuals with ASD [58,59]. Second, the decreased connectivity observed upon En2 treatment, combined to increased spine density, suggests that En2 over signaling could exaggerate the remodeling of synapses, a common phenotype across diverse ASD mouse models [60]. Third, as several ASD-linked genes (e.g. FMR1, TSC, PTEN and NF1), En upregulates mRNA translation, possibly the translation of a specific subset of mRNAs. Future work will permit to identify the primary molecular translational targets of En1 and En2, and to further characterize the En signaling pathway in dendrites in relation to both dendritic growth and ASDs.

## Supporting information

S1 Appendix Supplementary information. (PDF)

S1 Fig Validation of RT-qPCR experiments. (A, B), RT-qPCR experiments using Engrailed primers validated in En1 and En2 KO ([27], primers #2) confirm the developmental time course of Engrailed mRNA expression observed in the hippocampus by using primers #1 (see Material & Methods). (C) Comparison of Engrailed Ct values and fold change in expression levels (2^-ΔΔCT) obtained from hippocampal and cerebellar tissues. Hippocampal samples are highlighted in grey. Note that there are no significant differences between values normalized with HPRT or GAPDH or between values from male (M) or female (F). W, weeks, hipp, hippocampus, Cb, cerebellum.
(TIF)

S2 Fig Effect of Engrailed mutants on dendritic complexity. Additional analysis of dendritic complexity of glutamatergic cells (A-C) or GABAergic cells (D-F) by using En proteins (as in Fig 2) and En mutants (En1SR and En2SR) that have lost their cellular internalization capability [34]. Values are the mean +/- s.e.m. of about 20 measures from one experiment. Kruskal-Wallis test in A-C (****, p< 0.0001), and one-way ANOVA in D-F (****, p< 0.0001), followed respectively by a Dunn's Multiple Comparison Test and a Tukey’s Multiple Comparison Test. *, p<0.05; **, p<0.01; ***, p<0.001; ****, p<0.0001. Detailed P-values are given in Supplementary information.
(TIF)

S3 Fig En1 control spine density *in vivo*. (A), examples of images obtained after Golgi staining of mice hippocampi. Shown are apical and basal dendrites of wild type (wt) and *En1*^+/-^ mature mice (see quantification in B). (B, C) comparison of spine density (per μm) in WT and *En1*^*+/-*^ adult mice (B) or WT and *En2* KO adult mice (C). In B, ****, p<0.0001 (t-test). In C, *p = 0.0164, (Mann Whitney test). Values are the mean +/- s.e.m. of spine densities measured from a total of about 25–50 dendritic segments imaged from 3 mice per genotype. Bar in A, 5μm.
(TIF)

S4 Fig Engrailed increases the number of inhibitory synapses. (Top) examples of EGFP-labelled pyramidal cell dendrites immunostained with VIAAT; cells were either untreated (cont) or treated with either En1 or En2. (Bottom) quantification of the density of VIAAT-immunofluorescent clusters along dendrites of pyramidal cells in absence of treatment (cont) or after treatments with either En1, En2 or En1SR. Kruskal-Wallis test (*, p = 0.02) followed by Dunn's Multiple Comparison Test: cont vs En1, *p = 0.0117. Values are the mean +/- s.e.m. of 80 measures from 2 independent experiments. Bar, 5μm.
(TIF)

S5 Fig Image analysis. (A), Segmentation of synaptic area from confocal images of hippocampal neurons double stained for vGlut1 and PSD95. Sample of a maximal projection obtained from a z stack, and (B) the same picture exhibiting the synaptic areas obtained after detection and analysis of overlapping red and green objects. In blue, in B, is the user-defined selection delimiting the region of interest corresponding to the dendritic area. (C), zoom-in view of the area labelled by the white box in A, showing the synaptic density (Sd) analysis of the original stack using the Imaris software. This Sd model permits an easy visualization of the contacts between objects. (D), the same Sd representation as in C plus the outlines of synaptic areas detected by our program in B. Note the accuracy of the detection. (E, F) Analysis of arborization of hippocampal cells immunofluorescent for MAP2. In E, a glutamatergic neuron exhibiting MAP2 labelling. Soma area was manually highlighted and smoothed before automated image analysis. The picture shows original image and the vector detected objects (soma edge, red; master segments, orange; junctions, red surrounded by blue; master junctions, blue surrounded by red, and branches in green, see zoom inset in F. (F), Zoomed-in area coming from the selected area in panel E exhibiting segments (magenta), junctions (blue), and branches (green). Panel G shows residual structure after pruning the tree of panel F. The segments and junctions detected in this residual tree are called master segments and master junctions (i.e. nodes). Bar in A = 5μm, bar applies to B; in C = 1 μm, bar applies to D.
(TIF)
