# Supplementary material for: Increased signaling by the autism-related Engrailed-2 protein enhances dendritic branching and spine density, alters synaptic structural matching, and exaggerates protein synthesis
Source: PLoS One. 2017 Aug 15;12(8):e0181350. doi: 10.1371/journal.pone.0181350 (PMC5557355; doi:10.1371/journal.pone.0181350)
Supplement: S1 Appendix — (PDF) [file pone.0181350.s001.pdf]

Supplementary information

ImageJ routine for the acquisition of vGlut/PSD95 clusters

Image pre-treatment

Objects detection and analysis were performed after several pre-treatments allowing strong deblurring and denoising to get sharp and noise free details. Deblurring was performed by making a high pass filter from the mean resulting image of two unsharp masks (sigma 3 and 10) [4]. Noise was removed by replacing outlier pixels by the median of the pixels in the surrounding if they deviate from the median by more than a user-defined threshold value. Objects were then brought in a same range of intensity by applying an edge Sobel detection followed by a dilation.

Segmentation

Segmentation was performed by thresholding with a value optimized for each image, returning structures whose maximum size is n fold the mean size of the objects to analyze. "n" is set by the user according to the sampling during the laser scanning. The sub-segmentation of the aggregates was then performed by using a watershed operation [5]. A final sorting of the segmented objects was performed by using a ratio signal/noise (ratio S/N) threshold, defined as follows: ratio S/N = Max object / {Min outline + (0.5 * sd outline values) }. "Max object" is the maximum value got in an object. "Min outline" is the lowest value encounter in the outline of an object, and "sd outline values" is the standard deviation calculated from the outline pixels. Selected binary objects were then converted into vector objects (overlays).

Objects overlapping analysis

Vector objects got on the two channels corresponding to vGlut and PSD95 were submitted to an overlapping analysis into user selections delimiting axonal or dendritic edges. Red (PSD95) and green (vGlut) juxtaposed objects were considered as synaptic elements when their apparent edges overlapped more than 60 pixels (about 0.25µm^2). The program can analyze automatically batch of images and returns Excel like statistical tables of results [7].

References:

[1] Rasband WS (1997–2013) ImageJ website, U. S. National Institutes of Health, Bethesda, Maryland, USA. Available: http://imagejnihgov/ij/ Accessed 2012 August 26.

[2] Griffa A, Garin N, Sage D (2010) "Comparison of Deconvolution Software in 3D Microscopy., A User Point of View" Part I and Part II. GIT Imaging & Microscopy 1: 43–45.

[3] Vonesch C, Unser M (2008) A fast thresholded landweber algorithm for wavelet- regularized multidimensional deconvolution. IEEE Trans Image Process 17: 539–549.

[4] Ferreira T and Rasband W. (2013) ImageJ documentation, section 29.11.8 available at the http://rsb.info.nih.gov/ij/docs/guide/146-29.html#toc-Subsection-29.11.

[5] Ferreira T and Rasband W. (2013) ImageJ documentation, section 29.4 available at the http://rsb.info.nih.gov/ij/docs/guide/146-29.html#toc-Subsection-29.4.

[6] Carpentier G. (2013). Sort Selections in Overlays available at the http://rsb.info.nih.gov/ij/macros/Sort-Selections-in-Overlays.txt.

[7] Carpentier G. (2007). CustomTabStatFromResults available at the http://rsb.info.nih.gov/ij/macros/CustomTabStatFromResults.txt.

ImageJ routine for the analysis of dendritic morphology

Automated analysis of the neuron consists in three steps; detection of the soma edges, detection of the binary skeleton fitting to the neuritic tree and analysis of this tree by a customized version of the « Angiogenesis Analyzer » [1], a tool programmed for the ImageJ software [2]. Soma detection and binary skeletonizing can be summarized as follows: noise and background of initial image were removed respectively by Gaussian convolution with a sigma of 1.5 and a 2D so called « rolling ball filter » with a radius of 10 pixels. Thresholding by the « Otsu » [3] method returns the segmented image « A ». The soma detection was performed by using a FFT band pass filter on the initial image, using a size filtering in order of the mean diameter of the soma. A thresholding according to the « MaxEntropy » method [4] was then performed after background removal by subtracting the mean value of the image histogram. Edge of objects whose size corresponds to a soma was drawn and

emptied in the binary Image « A ». Skeletonizing the result of this step gave the binary skeleton then submitted to the modified « Angiogenesis Analyzer » for quantification of the tree: briefly, a first step deduced junctions, corresponding to bifurcation, branches (elements limited by one extremity and one junction) and segments (elements limited by two junctions), from the skeleton (Figures S4E and S4F). Pruning of this modelled tree resulted in a tree in which every branches was removed. Junctions and segments detected in this residual tree are called master segments and master junctions (Figures S4E and S4G). Measurement of these modelled structures allowed quantification of the neuronal structure.

References:

[1] Carpentier, G., 2012. Angiogenesis analyzer for ImageJ available at on the ImageJ website at the http://rsb.info.nih.gov/ij/macros/toolsets/Angiogenesis%20Analyzer.txt.

[2] Rasband, W.S., ImageJ, U. S. National Institutes of Health, Bethesda, Maryland, USA, http://imagej.nih.gov/ij/, 1997-2015.

[3] Otsu, N (1979), "A threshold selection method from gray-level histograms", IEEE Trans. Sys., Man., Cyber. 9: 62-66, doi:10.1109/TSMC.1979.4310076

[4] Kapur, JN; Sahoo, PK & Wong, ACK (1985), "A New Method for Gray-Level Picture Thresholding Using the Entropy of the Histogram", Graphical Models and Image Processing 29(3): 273-285

Linearity of the RT-qPCR primers (for Fig 1 and S1 Fig)

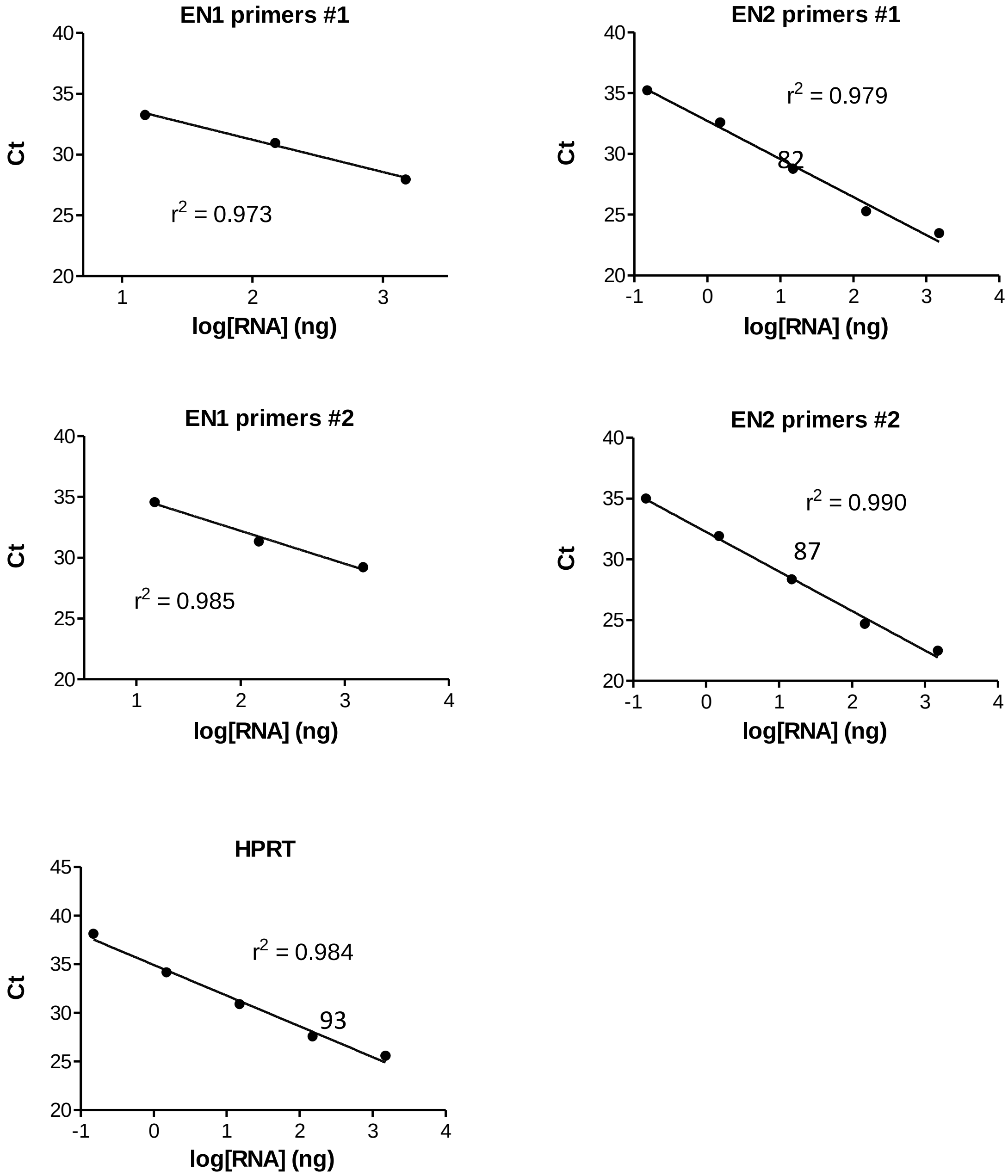

Multiple synaptic contacts (for Fig 4)

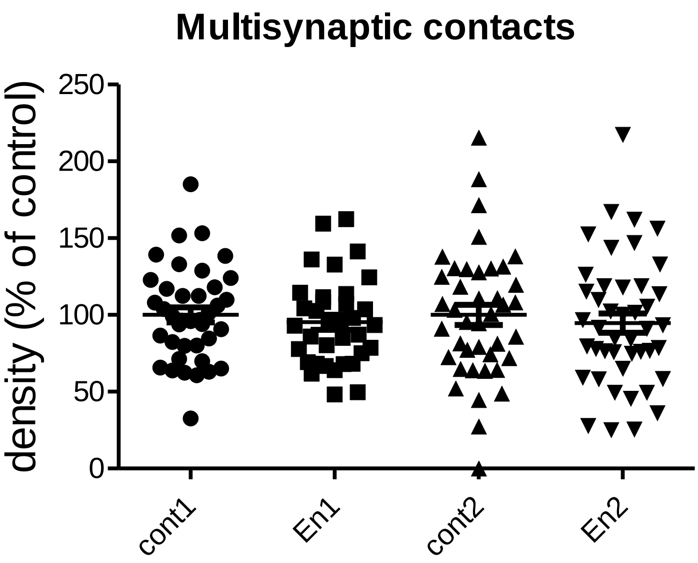

Detailed statistics of figures

Fig1A: Kruskal-Wallis test, *** p=0.0006. Dunn's Multiple Comparison Test: E16 vs 8W, **p=0.0012; P0 vs 8W, *p=0.0135.

Fig1B: Kruskal-Wallis test, **** p< 0.0001. Dunn's Multiple Comparison Test: E16 vs P0, **p=0.0051; E16 vs P5, *p=0.0310; P0 vs 8W, **p=0.0053, P0 vs 12W, **p=0.0017; P5 vs 8W, *p=0.0366; P5 vs 12W, *p=0.0154.

Fig1C: Mann Whitney test, ** p=0.004.

Fig1D: Mann Whitney test, p=0.1548.

Fig2B: Kruskal-Wallis test, **** p< 0.0001. Dunn's Multiple Comparison Test: cont-GLU vs En1-GLU, ***p=0.0002 ; cont-GLU vs En2-GLU, ***p=0.0008.

Fig2C: Kruskal-Wallis test, ***p=0.0006. Dunn's Multiple Comparison Test: cont-GLU vs En1-GLU, ***p=0.0007 ; cont-GLU vs En2-GLU, *p=0.0129.

Fig2D: Kruskal-Wallis test, **** p< 0.0001. Dunn's Multiple Comparison Test: cont-GLU vs En1-GLU, **p= 0.0012; cont-GLU vs En2-GLU, ****p<0.0001.

Fig2E: Kruskal-Wallis test, *** p=0.0002. Dunn's Multiple Comparison Test: cont-GABA vs En2-GABA, **p=0.0099; En1-GABA vs En2-GABA, ***p=0.0002.

Fig2F: Kruskal-Wallis test, **** p< 0.0001. Dunn's Multiple Comparison Test: cont-GABA vs En2-GABA, **p=0.0014; En1-GABA vs En2-GABA, ***p=0.0001.

Fig2G : Kruskal-Wallis test, **** p< 0.0001. Dunn's Multiple Comparison Test: cont-GABA vs En2-GABA, ****p<0001; En1-GABA vs En2-GABA, ****p<0001.

Fig3B: Kruskal-Wallis test, **** p< 0.0001. Dunn's Multiple Comparison Test: cont vs En1, **** p< 0.0001, cont vs En2, *p=.0.0166.

Fig3C: Kruskal-Wallis test, *** p=0.0001. Dunn's Multiple Comparison Test: cont vs En1, ***p=0.0007 ; En1 vs En1SR, **p=0.0014

Fig3D: Branched spines, t-test (one-tailed), cont vs En1, *** p=0.0004.

Fig3E: Stubby spines, t-test (one-tailed), cont vs En, **** p< 0.0001; Mushroom spines, Mann Whitney test, cont vs En, p=0.1874; Thin spines, t-test (one-tailed), cont vs En, *** p=0.0002.

Fig3F: Kolmogorov-Smirnov test on two samples (http://www.physics.csbsju.edu/stats/KS-test.html): stubby spine neck (KS test, D=0.159, p<0.0001), thin spine lengh (KS test, D=0.06, p=0.054), and mushroom spine volume (KS test, D=0.119, p=0.01).

Fig4C: Kruskal-Wallis test, ** p= 0.0099. Dunn's Multiple Comparison Test: cont1-Vglut vs En2-Vglut, *p=0.0368 ; cont2-Vglut vs En2-Vglut, *p=0.0158 ; cont1-PSD vs En2-PSD, *p=0.0432 ; cont2-PSD vs En2-PSD, **p=0.0052.

Fig4D: Kruskal-Wallis test, *** p= 0.0001. Dunn's Multiple Comparison Test: cont1-Vglut vs En2-Vglut, ***p=0.0009 ; En1-Vglut vs En2-Vglut, **p=0.0015 ; cont2-Vglut vs En2-Vglut, **p=0.0052 ; cont1-PSD vs En2-PSD, **p=0.0012 ; En1-PSD vs En2-PSD, ***p=0.0004 ; cont2-PSD vs En2-PSD, **p=0.0082.

Fig4E: Kruskal-Wallis test, *** p= 0.0001. Dunn's Multiple Comparison Test: cont1-over vs En2-over, **p=0.0012; En1-over vs En2-over, ***p=0.0007; cont2-over vs En2-over, **p=0.0068.

Fig5A: Kolmogorov-Smirnov test on two samples (http://www.physics.csbsju.edu/stats/KS-test.html): cont-PSD vs En1-PSD, D= 0.072, p=0.178; cont-Vglut vs En1-Vglut, D=0.084, p=0.055.

Fig5B: Kolmogorov-Smirnov test on two samples: cont-PSD vs En2-PSD, D= 0.183, p<0.0001; cont-Vglut vs En2-Vglut, D=0.055, p=0.568.

Fig5C: Kolmogorov-Smirnov test on two samples: cont-overlap vs En1-overlap, D=0.074, p=0.208.

Fig5D: Kolmogorov-Smirnov test on two samples: cont-overlap vs En2-overlap, D=0.112, p=0.047.

Fig6C: One-way ANOVA test, **** p< 0.0001. Tukey's Multiple Comparison Test: cont vs En1, **p=0.0026 ; cont vs En2, *p=0.0316 ; En1 vs En1+rapa, **p=0.0035 ; En2 vs En2+rapa, ***p=0.0003.

Fig6D: Kruskal-Wallis test, **** p< 0.0001. Dunn's Multiple Comparison Test: cont vs anisomycin, **** p< 0.0001; cont vs puromycin, **** p< 0.0001; anisomycin vs puromycin, **p=0.0011.

Fig6E: One-way ANOVA test, **** p< 0.0001. Turkey's Multiple Comparison Test: cont vs En1, *p=0.0123 ; cont vs En2, *p=0.0429 ; En1 vs En1+rapa, **** p< 0.0001; En2 vs En2+rapa, **** p< 0.0001.

FigS1A: Kruskal-Wallis test, ** p=0.0033. Dunn's Multiple Comparison Test: P0 vs 8W, *p=0.0101.

FigS1B: Kruskal-Wallis test, * p=0.0193. Dunn's Multiple Comparison Test: E16 vs P5, *p=0.0183.

FigS2A: Kruskal-Wallis test, **** p< 0.0001. Dunn's Multiple Comparison Test: no treatment (NT) vs En1SR, *p=0.0321; NT vs En1, **p=0.0024; NT vs En2, **** p< 0.0001; En2SR vs En2, **p=0.0061.

FigS2B: One-way ANOVA test, **** p< 0.0001. Turkey's Multiple Comparison Test: no treatment (NT) vs En1SR, *p=0.0132; NT vs En1, ***p=0.0005; NT vs En2, **** p< 0.0001; En2SR vs En1, *p=0.0183 ; En2SR vs En2, ***p=0.0002.

FigS2C: Kruskal-Wallis test, **** p< 0.0001. Dunn's Multiple Comparison Test: no treatment (NT) vs En1, **p=0.0069; NT vs En2, **** p< 0.0001; En2SR vs En2, **p=0.0012.

FigS2D: One-way ANOVA test, **** p< 0.0001. Turkey's Multiple Comparison Test: no treatment (NT) vs En2, **** p< 0.0001; En2SR vs En2, **** p< 0.0001.

FigS2E: One-way ANOVA test, **** p< 0.0001. Turkey's Multiple Comparison Test: no treatment (NT) vs En2, **** p< 0.0001; En2SR vs En2, **** p< 0.0001.

FigS2F: One-way ANOVA test, **** p< 0.0001. Turkey's Multiple Comparison Test: no treatment (NT) vs En2, *** p= 0.0001; En2SR vs En2, **** p< 0.0001.

FigS3B: Apical spine density, t-test, ****p<0.0001.

FigS3C: Basal spine density, Mann-Whitney test, *p=0.0164.

FigS4: Kruskal-Wallis test, * p=0.02. Dunn's Multiple Comparison Test: cont vs En1, *p=0.0117

Detailed dataset

Dataset are available at https://figshare.com/s/06a7d2a6d5db699dfaf1
