# Supplementary figures and images for: Increased signaling by the autism-related Engrailed-2 protein enhances dendritic branching and spine density, alters synaptic structural matching, and exaggerates protein synthesis
Source: PLoS One. 2017 Aug 15;12(8):e0181350. doi: 10.1371/journal.pone.0181350 (PMC5557355; doi:10.1371/journal.pone.0181350)

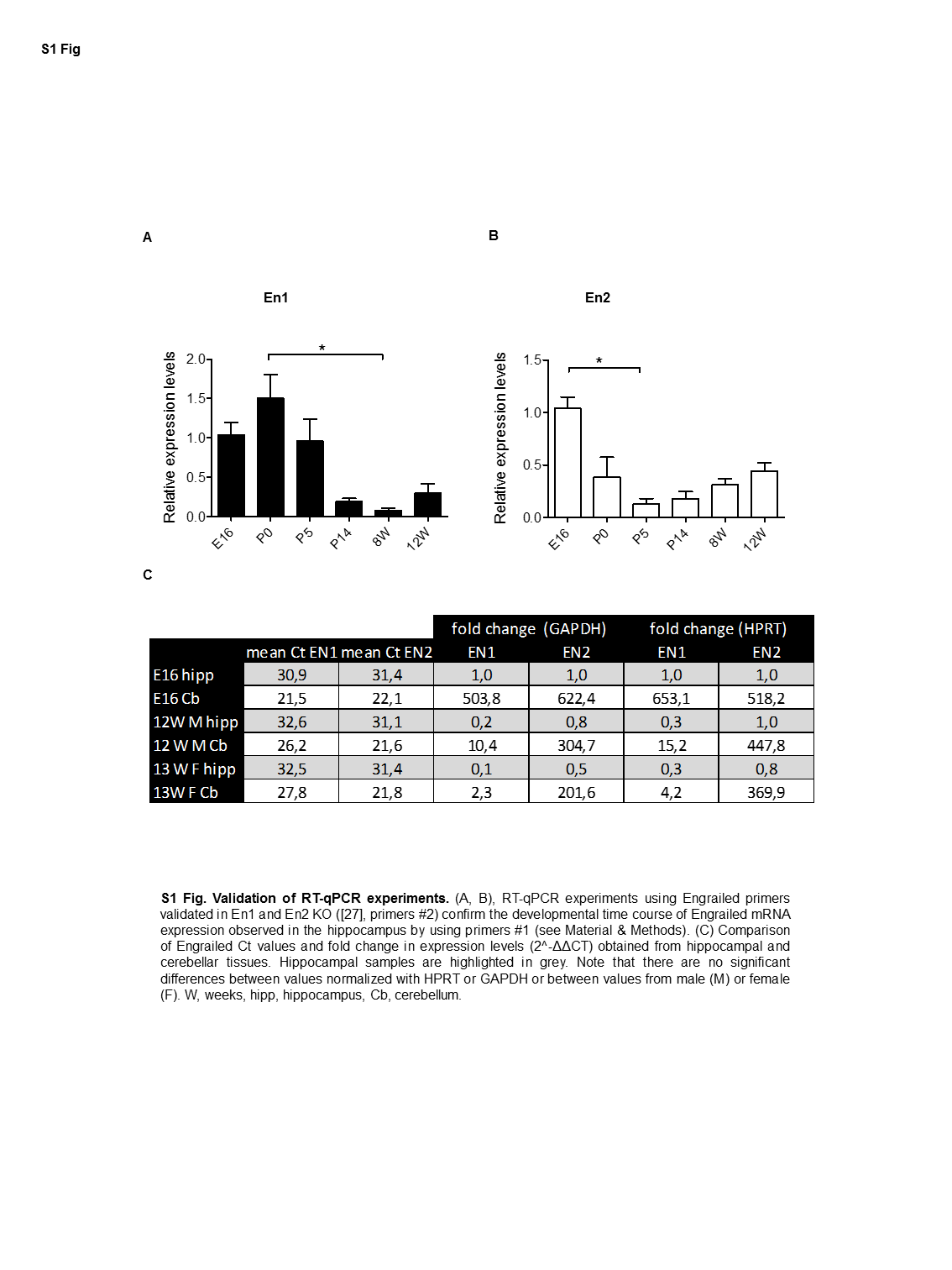

Supplement: S1 Fig — (A, B), RT-qPCR experiments using Engrailed primers validated in En1 and En2 KO ([27], primers #2) confirm the developmental time course of Engrailed mRNA expression observed in the hippocampus by using primers #1 (see Material & Methods). (C) Comparison of Engrailed Ct values and fold change in expression levels (2^-ΔΔCT) obtained from hippocampal and cerebellar tissues. Hippocampal samples are highlighted in grey. Note that there are no significant differences between values normalized with HPRT or GAPDH or between values from male (M) or female (F). W, weeks, hipp, hippocampus, Cb, cerebellum. (TIF) [file pone.0181350.s002.tif]

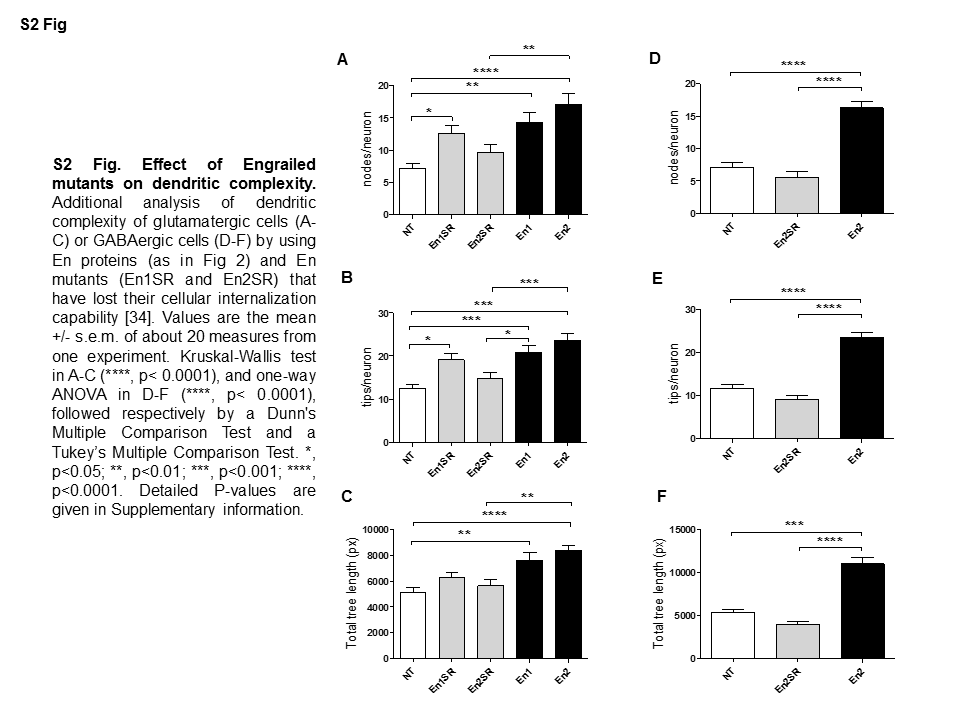

Supplement: S2 Fig — Additional analysis of dendritic complexity of glutamatergic cells (A-C) or GABAergic cells (D-F) by using En proteins (as in Fig 2) and En mutants (En1SR and En2SR) that have lost their cellular internalization capability [34]. Values are the mean +/- s.e.m. of about 20 measures from one experiment. Kruskal-Wallis test in A-C (****, p< 0.0001), and one-way ANOVA in D-F (****, p< 0.0001), followed respectively by a Dunn's Multiple Comparison Test and a Tukey’s Multiple Comparison Test. *, p<0.05; **, p<0.01; ***, p<0.001; ****, p<0.0001. Detailed P-values are given in Supplementary information. (TIF) [file pone.0181350.s003.tif]

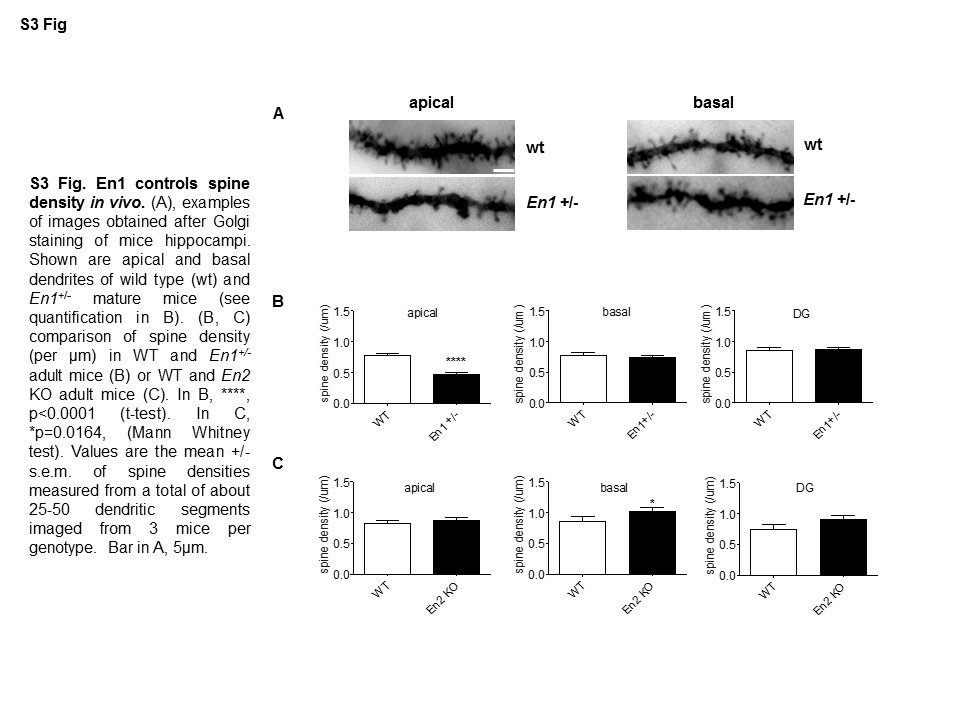

Supplement: S3 Fig — (A), examples of images obtained after Golgi staining of mice hippocampi. Shown are apical and basal dendrites of wild type (wt) and En1+/- mature mice (see quantification in B). (B, C) comparison of spine density (per μm) in WT and En1+/- adult mice (B) or WT and En2 KO adult mice (C). In B, ****, p<0.0001 (t-test). In C, *p = 0.0164, (Mann Whitney test). Values are the mean +/- s.e.m. of spine densities measured from a total of about 25–50 dendritic segments imaged from 3 mice per genotype. Bar in A, 5μm. (TIF) [file pone.0181350.s004.tif]

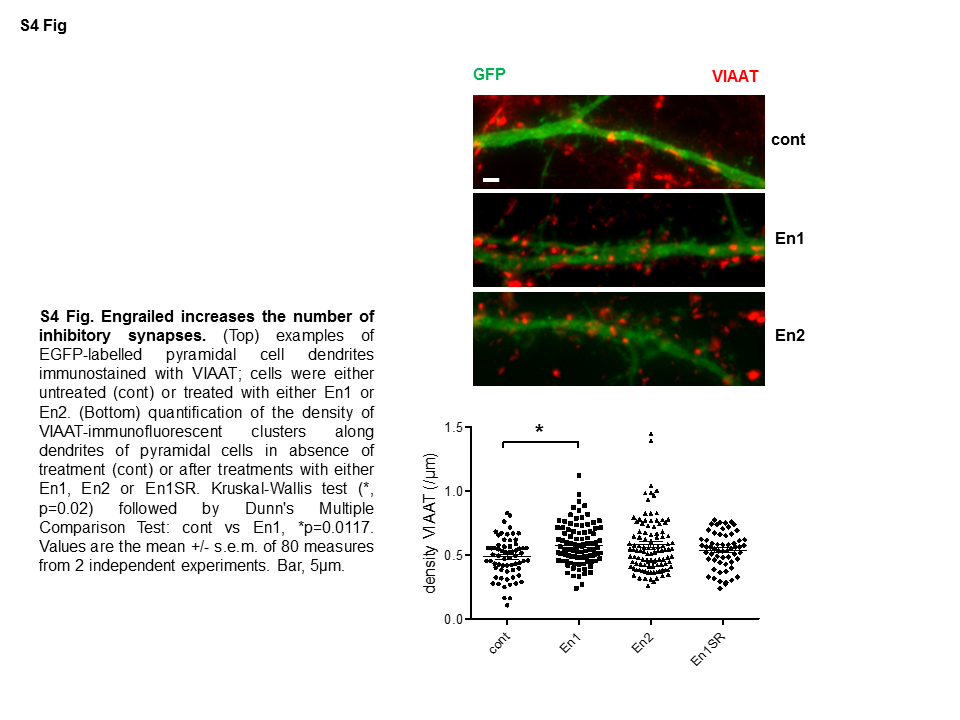

Supplement: S4 Fig — (Top) examples of EGFP-labelled pyramidal cell dendrites immunostained with VIAAT; cells were either untreated (cont) or treated with either En1 or En2. (Bottom) quantification of the density of VIAAT-immunofluorescent clusters along dendrites of pyramidal cells in absence of treatment (cont) or after treatments with either En1, En2 or En1SR. Kruskal-Wallis test (*, p = 0.02) followed by Dunn's Multiple Comparison Test: cont vs En1, *p = 0.0117. Values are the mean +/- s.e.m. of 80 measures from 2 independent experiments. Bar, 5μm. (TIF) [file pone.0181350.s005.tif]

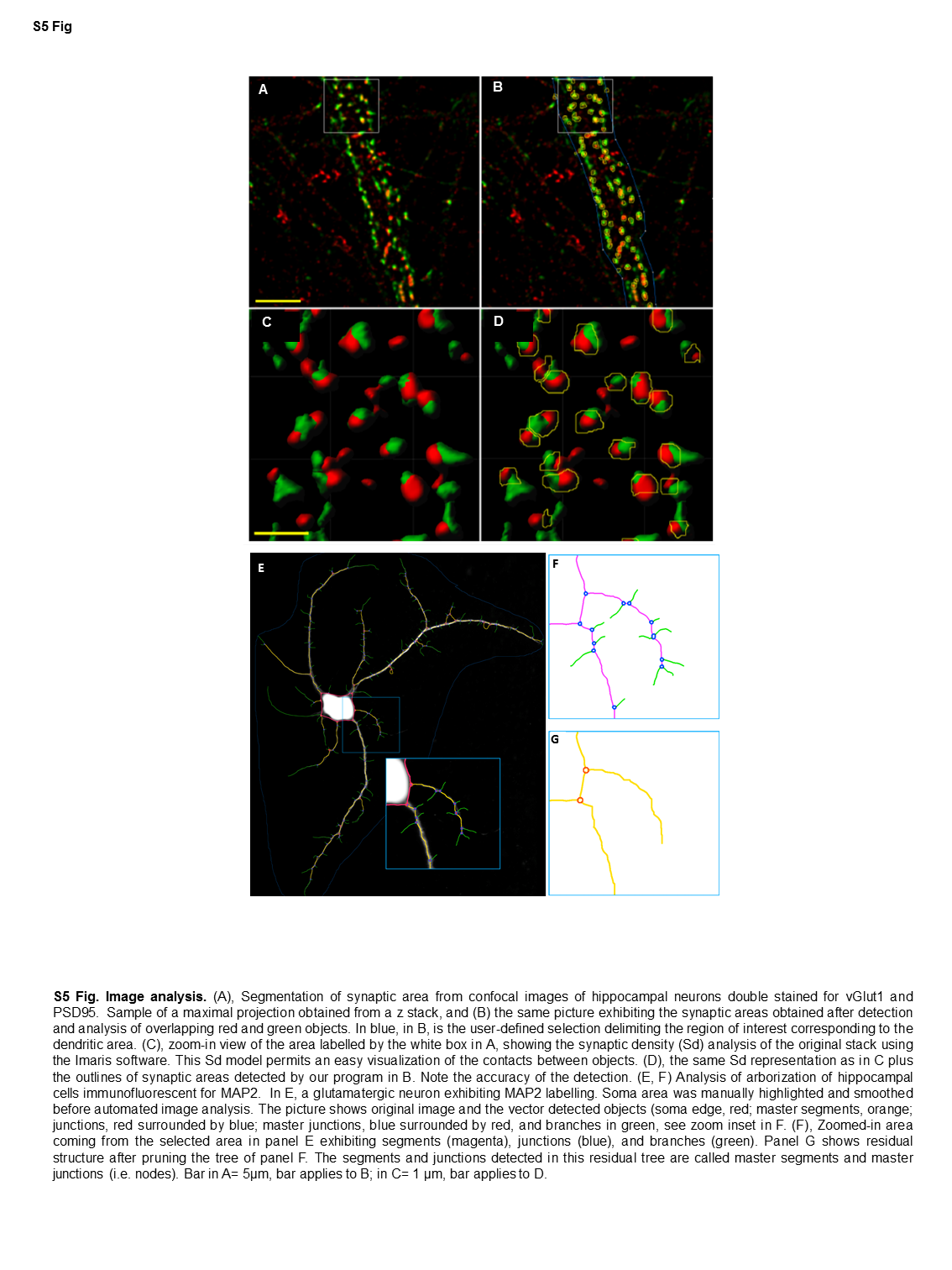

Supplement: S5 Fig — (A), Segmentation of synaptic area from confocal images of hippocampal neurons double stained for vGlut1 and PSD95. Sample of a maximal projection obtained from a z stack, and (B) the same picture exhibiting the synaptic areas obtained after detection and analysis of overlapping red and green objects. In blue, in B, is the user-defined selection delimiting the region of interest corresponding to the dendritic area. (C), zoom-in view of the area labelled by the white box in A, showing the synaptic density (Sd) analysis of the original stack using the Imaris software. This Sd model permits an easy visualization of the contacts between objects. (D), the same Sd representation as in C plus the outlines of synaptic areas detected by our program in B. Note the accuracy of the detection. (E, F) Analysis of arborization of hippocampal cells immunofluorescent for MAP2. In E, a glutamatergic neuron exhibiting MAP2 labelling. Soma area was manually highlighted and smoothed before automated image analysis. The picture shows original image and the vector detected objects (soma edge, red; master segments, orange; junctions, red surrounded by blue; master junctions, blue surrounded by red, and branches in green, see zoom inset in F. (F), Zoomed-in area coming from the selected area in panel E exhibiting segments (magenta), junctions (blue), and branches (green). Panel G shows residual structure after pruning the tree of panel F. The segments and junctions detected in this residual tree are called master segments and master junctions (i.e. nodes). Bar in A = 5μm, bar applies to B; in C = 1 μm, bar applies to D. (TIF) [file pone.0181350.s006.tif]
